# The prevalence of bronchodilator responsiveness of the small airway (using mid-maximal expiratory flow) in COPD - a retrospective study

**DOI:** 10.1186/s12890-022-02235-0

**Published:** 2022-12-30

**Authors:** Nowaf Y. Alobaidi, Mohammed A. Almeshari, James A. Stockley, Robert A. Stockley, Elizabeth Sapey

**Affiliations:** 1 Birmingham Acute Care Research Group, Institute of Inflammation and Ageing, University of Birmingham, Birmingham, B15 2TT UK; 2 Respiratory Therapy Department, King Saud Bin Abdulaziz University for Health Sciences, Alahsa, Saudi Arabia; 3 Rehabilitation Health Sciences Department, College of Applied Medical Sciences, King Saud University, Riyadh, Saudi Arabia; 4 Lung Function & Sleep Department, Respiratory Medicine, University Hospitals Birmingham NHS Foundation Trust, Queen Elizabeth Hospital Birmingham, Birmingham, UK; 5 Department of Respiratory Medicine, University Hospitals Birmingham NHS Foundation Trust, Edgbaston, Birmingham, UK; 6 Acute Medicine, University Hospitals Birmingham NHS Foundation Trust, Edgbaston, Birmingham, B15 2GW UK

**Keywords:** COPD, Spirometry, Bronchodilator responsiveness, MMEF, FEV_1_

## Abstract

**Background:**

Bronchodilator responsiveness (BDR) using FEV_1_ is often utilised to separate COPD patients from asthmatics, although it can be present in some COPD patients. With the advent of treatments with distal airway deposition, BDR in the small airways (SA) may be of value in the management of COPD. We aimed to identify the prevalence of BDR in the SA, utilizing maximal mid-expiratory flow (MMEF) as a measure of SA. We further evaluated the prevalence of BDR in MMEF with and without BDR in FEV_1_ and its association with baseline demographics, including conventional airflow obstruction severity and smoking history.

**Methods:**

Lung function data of ever-smoking COPD patients were retrospectively analysed. BDR was evaluated 20 min after administering 2.5 mg of salbutamol via jet nebulizer. Increase in percent change of ≥ 12% and absolute change of ≥ 200 ml was used to define a BDR in FEV_1_, whereas an increase percent change of MMEF ≥ 30% was used to define a BDR in MMEF. Patients were classified as one of three groups according to BDR levels: group 1 (BDR in MMEF and FEV_1_), group 2 (BDR in MMEF alone) and group 3 (no BDR in either measure).

**Result:**

BDR in MMEF was present in 59.2% of the patients. Of note, BDR in MMEF was present in all patients with BDR in FEV_1_ (group 1) but also in 37.9% of the patients without BDR in FEV_1_ (group 2). Patients in group 1 were younger than in groups 2 and 3. BMI was higher in group 1 than in group 3. Baseline FEV_1_% predicted and FVC % predicted were also higher in groups 1 and 2 than in group 3.

**Conclusion:**

BDR in the SA (evaluated by MMEF) is common in COPD, and it is also feature seen in all patients with BDR in FEV_1_. Even in the absence of BDR in FEV_1_, BDR in MMEF is detected in some patients with COPD, potentially identifying a subgroup of patients who may benefit from different treatment strategies.

**Supplementary Information:**

The online version contains supplementary material available at 10.1186/s12890-022-02235-0.

## Background

Chronic Obstructive Pulmonary Disease (COPD) is a chronic inflammatory disease characterised by poorly reversible airflow limitation [1]. Bronchodilator responsiveness (BDR) can be used to help distinguish between COPD and asthma [1–4] and is a valuable indicator of prognosis in asthma [5], although it is increasingly recognised that some patients with COPD also display BDR [6–8]. The usefulness of BDR assessment in COPD remains unclear [9] but BDR in both the forced expiratory volume in the first second (FEV_1_) and the forced vital capacity (FVC) can identify subjects with less emphysema, more exacerbations and greater lung function decline but a lower mortality risk than subjects with no BDR [10].

The assessment of BDR usually depends on the measurement of the FEV_1_ although definitions of BDR can vary between guidelines [11]. A BDR is commonly defined by an increase in percent change of ≥ 12% and in absolute change of ≥ 200 ml [11, 12]. Studies have shown that vital capacity and inspiratory capacity can increase in the absence of a positive FEV_1_ response [13, 14], indicating that a BDR should not be determined by a single spirometric measure alone and additional physiological measures may be of value in understanding and managing COPD.

Physiological and pathological studies have demonstrated that small airways dysfunction (SAD) is an important feature of COPD [15–17]. Maximal Mid-Expiratory Flow (MMEF) is the most widely reported measure of small airway function and has become a valuable marker of SAD, especially in the detection of early stages of COPD [17–19].

A recent systematic review reported that studies exploring the use of tests of small airways (SA) in BDR assessment in COPD are few and involve small number of participants [20]. Despite this, the studies reported that BDR as defined by tests of SA could potentially identify specific groups of patients and suggested that, with the advent of ultra-fine particles inhalers, there may be specific treatment options for those with BDR detected in the SA [21, 22]. Further studies were suggested to determine the potential utility of tests of SA in defining BDR in COPD.

We hypothesised that a BDR within the SA (defined by a BDR in MMEF) would be common in COPD, seen in most COPD patients with a BDR defined by FEV_1,_ but also in some without BDR in FEV_1_. Moreover, we hypothesised that those with a BDR in the SA alone would not be distinguishable in terms of demography, smoking exposure or severity of disease from those without BDR in the SA. However, this might potentially identify a subgroup of COPD patients who may benefit from more peripheral bronchodilator deposition using ultra-fine particles inhalers and could, therefore, form a group where screening was warranted to identify a differently treatable characteristic.

To assess these hypotheses, we evaluated routinely collected data from COPD patients to determine the prevalence of BDR in MMEF. We further assessed the prevalence of BDR in MMEF with and without BDR in FEV_1_, and its association with baseline demography, airflow obstruction (AO) severity and smoking history.

## Methods

### Study design and setting

This was a retrospective study of historical lung function data from COPD patients who underwent pulmonary function testing at the University Hospitals Birmingham NHS Foundation Trust, UK. The study utilised data collected between January 2016 and April 2021. The study, and specifically the use of anonymised routinely collected health data was carried out in accordance with the Declaration of Helsinki and was approved by the Health Research Authority (HRA – project number 274729) and the South Birmingham Research Ethics Committee (Reference number 20/WM/0024). The HRA and South Birmingham Research Ethics Committee (Reference number 20/WM/0024) waived the need for informed consent as the study was retrospective. All methods were conducted in line with the HRA's guidelines and regulations.

### Eligibility criteria

The study included participants if they had the following:

- 1) Confirmed diagnosis of COPD (post-bronchodilator (BD) FEV_1_/FVC ratio < lower limit of normal; z-score = 1.645 or 0.70)
- 2) Aged 30 years and over
- 3) Ten or more pack-years history of smoking
- 4) Pre- and post-BD spirometric measures, including MMEF.
- Participants were excluded if they had COPD related to AATD, other factors which might alter the interpretation of lung function tests, a clinician defined history/diagnosis of other chronic lung diseases (such as asthma), or significant radiological bronchiectasis.

### Study measures

Patient demographics were collected, including age, sex, body mass index (BMI) and smoking history, which included current smoking status (ex- or current smokers), pack-years and years since quitting and long-term medications.

Pre- and post-BD lung function parameters were documented, including FEV_1_, FVC, FEV_1_/FVC, MMEF, MMEF corrected for lung volume (MMEF/FVC), forced expiratory volume exhaled in the first 3 s (FEV_3_) and FEV_3_/FVC. MMEF/FVC was obtained by dividing MMEF % predicted by FVC % predicted [23]. Using post-BD FEV_1_% predicted, we evaluated AO severity using the Global Initiative for Chronic Obstructive Lung Disease (GOLD) criteria [1]. Lung function parameters were obtained using the Ultima PF™ Pulmonary Lung Function System (Medical Graphics UK Ltd, Tewkesbury, UK), performed following the Association for Respiratory Technology and Physiology/British Thoracic Society guidelines [24]. In this study, predicted values for spirometric measures were obtained from the European Community for Steel and Coal [25]. The study used the formula from the Global Lung Function Initiative 2012 to calculate the LLN from the spirometric measures [26].

BDR was evaluated 20 min after the administration of 2.5 mg of salbutamol by a jet nebulizer.

BDR in FEV_1_ was defined according to American Thoracic Society/ European Respiratory Society criteria (increase of ≥ 12 in percent change (% change) and ≥ 200 ml) [11]. BDR in the SA was evaluated using MMEF, which was defined by an increase of ≥ 30 in % change, based on previous studies [27, 28].

In an initial analysis, COPD patients (defined using the LLN) were divided into two groups according to their BDR in MMEF:

- Patients with BDR in MMEF
- Patients without BDR in MMEF
  Using the pre-defined BDR definition of FEV_1_ and MMEF, COPD patients (defined using the LLN) were further classified into three groups according to their BDR features:
- Group 1 (BDR in FEV_1_ and MMEF),
- Group 2 (BDR in MMEF but not in FEV_1_),
- Group 3 (no BDR in either FEV_1_ or MMEF).

We further compared the prevalence of BDR in MMEF with and without BDR in FEV_1_ in COPD patients using FEV_1_/FVC < LLN (z-score -1.645) and the < 0.70.

### Statistical analysis

The Shapiro–Wilk normality test was used, which confirmed the data was non-normally distributed and median and interquartile was reported for all variables. The Kruskal–Wallis H was performed, and if it demonstrated statistical significance difference, pairwise comparisons using Dunn’s test were conducted to determine differences. Categorical variables were evaluated using Chi-square or Fisher’s exact test. Using the Bonferroni method, adjustment for *p*-values was made to account for multiple comparisons. IBM SPSS software was used for all statistical analyses.

## Results

### Participant’s selection

**Fig. 1:**
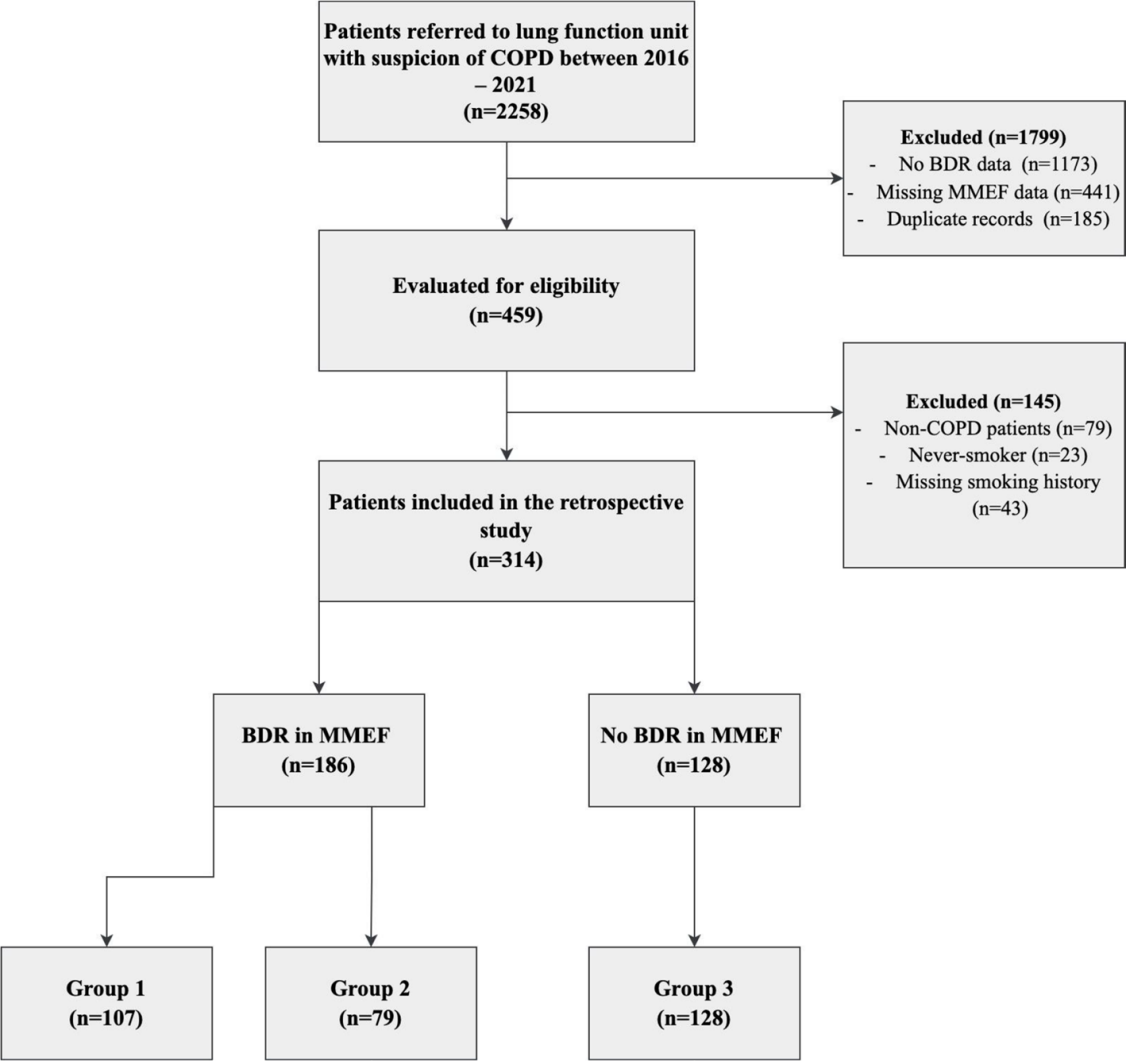
Flowchart of the study. Legend: This figure demonstrates the selection process for patients according to eligibility criteria. Group 1, those with BDR in FEV_1_ and MMEF; Group 2, those with BDR in MMEF alone; Group 3, those with no BDR in either FEV_1_ or MMEF. Abbreviations: COPD, chronic obstructive pulmonary disease; MMEF, maximal mid-expiratory flow; FEV_1_, forced expiratory volume in 1 s; BDR, bronchodilator response

There were 2285 lung function recordings taken for patients with COPD within the timeframe but after eligibility evaluation, 314 patients (using the LLN criteria) were included in the study (see Fig. 1 for a flow diagram providing details of reasons for exclusion). Initially, patients were grouped into those with BDR in MMEF (*n* = 186) and those without BDR in MMEF (*n* = 128). Including the BDR response in FEV_1_ with MMEF: BDR in FEV_1_ and MMEF (Group 1; *n* = 107), BDR in MMEF alone (Group 2; *n* = 79) and no BDR in either measure (Group 3; *n* = 128). All patients with a BDR in FEV_1_ had a BDR in MMEF. Using the 0.70 criteria, 353 patients were included (see supplementary figure). Of those, 206 patients had BDR in MMEF and 147 had no BDR in MMEF. The groups for the BDR in FEV_1_ and MMEF using the 0.70 criteria were as follow: (Group 1; *n* = 114), BDR in MMEF alone (Group 2; *n* = 92) and no BDR in either measure (Group 3; *n* = 147).

### Prevalence of BDR in MMEF

Using the LLN criteria, of the 312 included patients, 59.2% demonstrated a BDR in MMEF. Of whom, 57.5% had BDR in FEV_1_. Using the 0.70 criteria, 58.3% showed BDR in MMEF, and of those, 55.3 had BDR in FEV_1_.

### Prevalence of BDR in MMEF with and without BDR in FEV_1_

Grouping patients into those with BDR in FEV_1_ and MMEF; MMEF alone; or no BDR in either measure confirmed that all patients with a BDR in FEV_1_ had BDR in MMEF (group 1). Of those without BDR for FEV_1_, 37.98% (79/208) had a positive BDR in MMEF alone (group 2). Using the 0.70, BDR in MMEF was also prevalent in all COPD patients with BDR in FEV_1_ and seen in 38.5% (92/239) in the absence of BDR in FEV_1_ (see supplementary table 1).

### Baseline demographics

Baseline characteristics of the patients in each of the 3 groups (using the LLN criteria) are summarized in Table 1. Apart from age (younger patients in group 1 than in group 2 [median age 60 vs 67, *p* = 0.040] and group 3 [median age 60 vs 67, *p* = 0.003]) and BMI (higher in group 1 than in group 3 [median 27.77 vs 25.81, *p* = 0.015]), there were no differences in baseline demographics across groups.

**Table 1:** Baseline demographics across groups

Variable	Group 1 *n* = 107	Group 2 *n* = 79	Group 3 *n* = 128
**Age**	60 (54 – 71)^cd^	67 (56 – 73)	67 (58 – 74)
**Sex (n, %)**			
Male	71 (67)	40 (50.6)	67 (51.9)
Female	35 (33)	39 (49.4)	62 (48.1)
**BMI (kg/m**^**2**^**)**	27.75 (23.94 – 31.63)^d^	25.89 (22.77 – 31.22)	25.82 (22.13 – 29.69)
**Smoking status (n, %)**			
Current smoker	62 (58.5)	50 (63.3)	81 (62.8)
Ex-smoker	44 (41.5)	29 (36.7)	48 (37.2)
**Pack-years** ^**a**^	38 (25 – 52)	42 (26 – 54)	40 (25 – 57)
**Years quit** ^**a**^	7 (2 – 12)	12 (3 – 18)	10 (2 – 20)
**Medications (n, %)**			
SABA or SAMA	62 (58.5)	44 (55.7)	75 (58.1)
SABA + SAMA	0 (0)	1 (1.3)	2 (1.6)
LABA or LAMA	27 (25.5)	16 (20.3)	32 (24.8)
LABA + LAMA	1 (0.9)	2 (2.5)	3 (2.3)
ICS	5 (4.7)	7 (8.9)	6 (4.7)
LABA + ICS	12 (11.3)	10 (12.7)	18 (14)
LABA + LAMA + ICS	13 (12.3)	10 (12.7)	17 (13.2)

*Abbreviations: BMI* body mass index, *SABA* short-acting beta-2 agonist, *SAMA* short-acting muscarinic antagonist, ICS inhaled corticosteroid, *LABA* long-acting beta-2 agonist, *LAMA* long-acting muscarinic antagonist, *BDR* bronchodilator responsiveness, *FEV1* forced expiratory volume in one second, *MMEF* maximal mid-expiratory flow

Legend: Data are presented as median (IQR) or n (%). Group 1, BDR in FEV1 and MMEF; group 2, BDR in MMEF alone; group 3, no BDR in either measure.

^a^This was only assessed in ex-smokers

^b^Significantly different from group 1

^c^Significantly different from group 2

^d^Significantly different from group 3

### Lung function and bronchodilator response

**Fig. 2:**
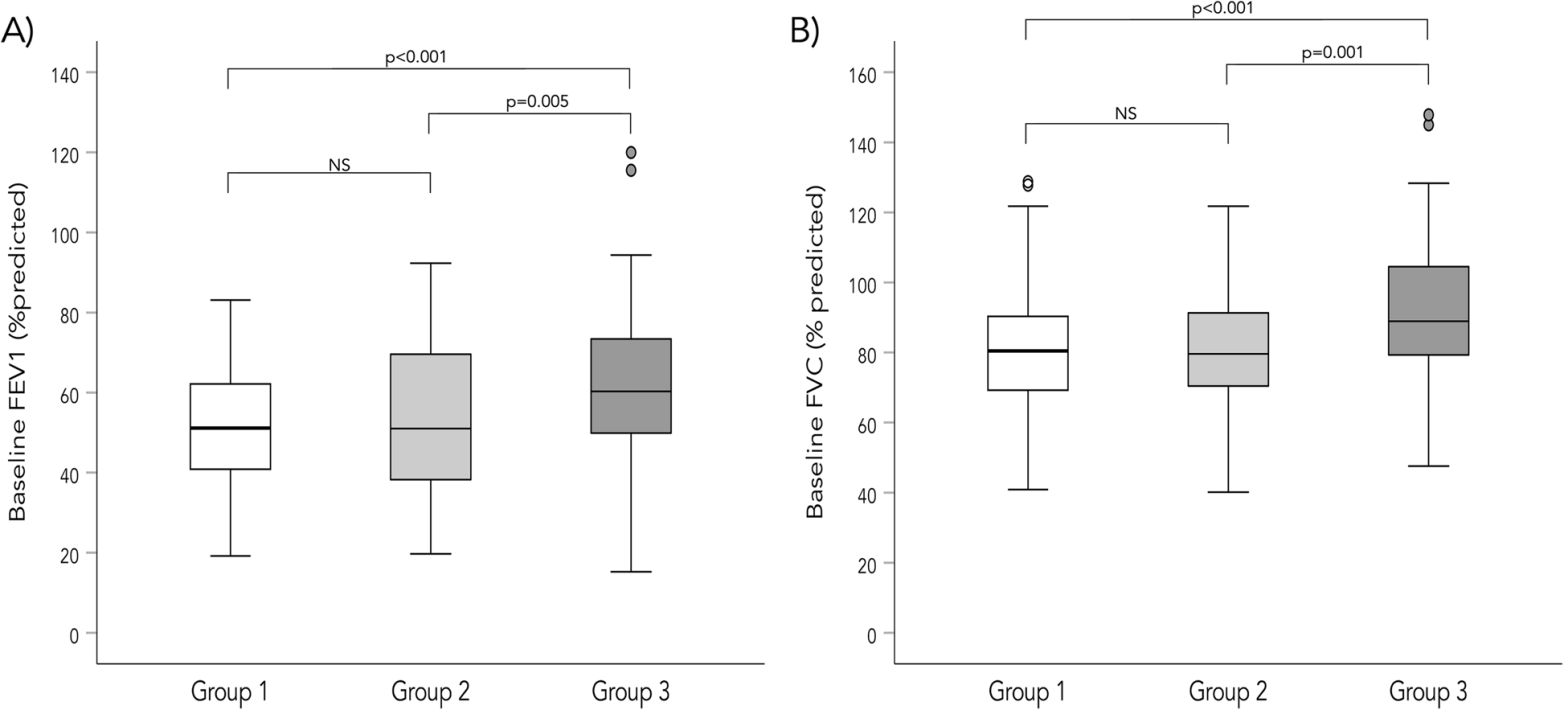
Baseline spirometric measures across groups. Legend: This figure demonstrates the distribution of baseline spirometric measures across groups. **A** Baseline FEV_1_% predicted. **B** Baseline FVC % predicted. Abbreviations: MMEF, maximal mid-expiratory flow; FEV_1_, forced expired volume in the first second; FVC, forced vital capacity; Group 1, BDR in FEV_1_ and MMEF; group 2, BDR in MMEF alone; group 3, no BDR in either measure

Table 2 describes the baseline spirometric measures, post-BD spirometric measures and the post-BD change for the BDR groups. In general, patients in groups 1 and 2 had lower baseline lung function measures (lower FEV_1_% predicted and lower FVC % predicted [*p* < 0.001 for both variables]) than group 3. The distribution of baseline FEV_1_% predicted and FVC % predicted groups are graphically shown in Fig. 2.

**Table 2:** Baseline and post-BD spirometric measures and post-BD changes across groups

Variable	Group 1 *n* = 107	Group 2 *n* = 79	Group 3 *n* = 128
**FEV**_**1**_			
Baseline (L)	1.50 (1.21 – 2.02)	1.22 (0.89 – 1.90)^ac^	1.47 (1.15 – 2.08)
Baseline (% predicted)	51.11 (40.82 – 62.13)	51.02 (38.02 – 70.66)	60.28 (49.85 – 73.40)^ab^
Post-BD (L)	1.86 (1.58 – 2.39)^bc^	1.37 (1.04 – 2.05)	1.57 (1.20 – 2.12)
Post-BD (% predicted)	63.67 (55.84 – 72.94)	56.12 (43.48 – 76.79)	62.16 (49.30 – 73.72)
Post-BD change (L)	0.33 (0.28 – 0.44)	0.16 (0.13 – 0.19)^a^	0.05 (0 – 0.10)^ab^
Post-BD change (% predicted)	11.29 (9.36 – 14.46)	6.12 (5.15 – 7.52)^a^	1.96 (0 – 3.60)^ab^
%Change of initial	21.34 (17.48 – 32.65)	11.51 (9.50 – 15.38)^a^	3.60 (0 – 6.43)^ab^
**FVC**			
Baseline (L)	3.17 (2.39 – 3.72)	2.64 (2.07 – 3.30)	3.03 (2.40 – 3.80)
Baseline (% predicted)	80.47 (69.25 – 90.31)	79.63 (69.68 – 91.45)	88.94 (79.33 – 104.54)^ab^
Post-BD (L)	3.67 (2.91 – 4.21)^bc^	3.05 (2.46 – 3.65)	3.12 (2.42 – 3.93)
Post-BD (% predicted)	91.56 (80.99 – 104.18)	89.77 (77.97 – 102.86)	92.28 (81.89 – 104.41)
Post-BD change (L)	0.47 (0.29 – 0.67)	0.33 (0.20 – 0.46)^a^	0.07 (-0.01 – 0.18)^ab^
Post-BD change (% predicted)	11.95 (7.53 – 18.62)	9.55 (6.06 – 13.51)^a^	1.84 (-0.51 – 5.17)^ab^
%Change of initial	16.06 (8.60 – 27.40)	11.97 (6.53 – 17.28)^a^	2.34 (-0.49 – 5.81)^ab^
**FEV**_**1**_**/FVC**			
Baseline (%)	52 (45 – 57)	52 (41 – 59)	53 (46 – 60)
Post-BD (%)	55 (49 – 61)	53 (41 – 59)	54 (44 – 60)
Post-BD change (%)	3 (0 – 6)^bc^	0 (-1 – 2)	0 (-2 – 2)
**MMEF**			
Baseline (L/s)	0.62 (0.47 – 0.77)^b^	0.45 (0.32 – 0.83)	0.57 (0.39 – 0.90)
Baseline (% predicted)	23.50 (18.80 – 29.67)	23.24 (13.84 – 37.52)	26.90 (18.94 – 36.85)
Post-BD (L/s)	1.12 (0.87 – 1.43)^bc^	0.64 (0.44 – 1.17)	0.59 (0.41 – 0.99)
Post-BD (% predicted)	43 (32.83 – 55.09)^bc^	34.48 (21.53 – 53.77)	28.14 (21.51 – 40.20)
Post-BD change (L/s)	0.47 (0.32 – 0.65)	0.21 (0.15 – 0.35)^a^	0.05 (0 – 0.10)^ab^
Post-BD change (% predicted)	17.91 (12.63 – 26.09)	10.49 (6.44 – 15.04)^a^	2.63 (0 – 4.56)^ab^
%Change of initial	76.24 (57.54 – 96.88)	42.34 (35.44 – 53.57)^a^	11.77 (0 – 20)^ab^
**MMEF/FVC**			
Baseline (%)	30.23 (25.27 – 38.15)	30.69 (20.45 – 40.54)	29.67 (22.54 – 42.22)
Post-BD (%)	47.54 (36.82 – 61.38)	41.15 (24.10 -52.97)^a^	31.34 (24.93 – 42.80)^ab^
Post-BD change (% predicted)	16.48 (10.52 – 21.70)	8.99 (5.14 – 12.54)^a^	2.13 (-0.39 – 4.22)^ab^
**FEV**_**3**_			
Baseline (L)	2.26 (1.89 – 2.87)	1.87 (1.42 – 2.58)^ac^	2.18 (1.74 – 2.88)
Post-BD (L)	2.74 (2.27 – 3.31)^bc^	2.06 (1.67 – 2.81)	2.28 (1.78 – 2.91)
Post-BD change (L)	0.42 (0.32 – 0.53)	0.22 (0.19 – 0.29)^a^	0.07 (0.01 – 0.12)^ab^
%Change of initial	17.92 (13.50 – 23.85)	10.99 (8.53 – 15.38)^a^	3.24 (0.68 – 5.19)^ab^
**FEV**_**3**_**/FVC**			
Baseline (%)	77.14 (71.92 – 81.57)	76.57 (64.02 – 82.19)	76.74 (70.03 – 83.07)
Post-BD (%)	79.28 (72.67 – 83.33)	77.84 (67.67 – 82.98)	76.84 (68.82 – 83.07)
Post-BD change (%)	1.51 (-0.98 – 3.89)^c^	0.10 (-3.04 – 3.32)	0.33 (-2.50 – 2.53)
**GOLD stages (n, %)**			
GOLD I	16 (15.1)	13 (16.5)	18 (14)
GOLD II	70 (66)^b^	35 (44.3)	78 (60.5)
GOLD III	19 (17.9)	25 (31.6)	28 (21.7)
GOLD IV	1 (0.9)	6 (7.6)	5 (3.9)

*Abbreviations*: *FEV*_*1*_ forced expiratory volume in one second, *FVC* forced vital capacity, *MMEF* maximal mid-expiratory flow, *FEV*_*3*_ forced expiratory volume in 3 s, *BDR* bronchodilator responsiveness, *BD* bronchodilator

Legend: Data are presented as either median (IQR) or n (%). Group 1, BDR in FEV_1_ and MMEF; group 2, BDR in MMEF alone; group 3, no BDR in either measure

^a^Significantly different from group 1

^b^Significantly different from group 2

^c^Significantly different from group 3

Groups 1 and 2 also had greater BDR (including FEV_1_, FVC, MMEF, MMEF/FVC and FEV_3_ [*p* < 0.001 for all variables]) than group 3, whereas patients in group 2 had less BDR than in group 1 (*p* < 0.001).

GOLD stages were not different across the three groups, except GOLD II which was less prevalent in group 2 than group 1.

## Discussion

The present study provides data on BDR using the commonly reported measures from spirometry (FEV_1_) and a widely recognised measure of SAD (MMEF) in ever-smoking COPD patients and emphasises three key points.

Firstly, BDR in the MMEF is common in COPD, detected in 59% of COPD patients in this cohort.

Secondly, BDR in MMEF is observed in all patients with BDR in FEV_1_, signifying that the change in MMEF represents a physiological improvement that likely reflects improvement in SA patency. However, comprehensive studies, including the assessment of static lung volumes and gas transfer, are needed to support this.

Thirdly, BDR in MMEF is also present in some patients without BDR in FEV_1_, indicating a potential utility of MMEF as an additional test in global lung BDR assessment. Here, the post-BD change (though crossing our threshold for BDR in MMEF) was less than that seen in those with BDR in both MMEF and FEV_1_, which may subsequently have less effect on FEV_1_ than seen when with greater improvement in MMEF. This highlights that bronchodilator therapy (particularly those targeting small airways) would likely benefit such patients even if no significant change was seen in FEV_1_. However, exploring this further requires prospective studies to determine whether the response in MMEF alone represents a detectable clinical effect.

Using the 0.70 cut-off to define COPD, we found a similar pattern in terms of overall prevalence findings compared to the use of LLN. The use of 0.70 demonstrated that BDR in MMEF is prevalent in all COPD patients with BDR in FEV_1_ and seen in some in the absence of BDR in FEV_1_. However, using the 0.70 resulted in a higher total number of participants who would not be classified with COPD whereas they would be using the LLN (*n* = 353 vs *n* = 312).

The present study found that baseline demographics, including age, smoking exposure and AO severity, were not different in those with BDR in MMEF alone and those without BDR in either measure, which is in line with the study hypothesis. This highlights that although BDR in the MMEF alone could not be predicted by any baseline demography, the MMEF changes seen in these patients indicate that these likely reflect a physiological improvement and could be of value as it may form a group with differently treatable traits. However, whether these improvements are of benefit to symptoms is currently unclear as such measures were not collected routinely and hence, requires further study for confirmation.

Grouping patients with BDR in MMEF into those with and without BDR in FEV_1_ was not related to baseline spirometric measures, except FEV_1_ and FVC % predicted, which were lower in those exhibiting MMEF BDR (groups 1 and 2) than those without BDR in either measure (group 3). The findings indicate that having a lower baseline FEV_1_ and FVC will likely increase the chances of patients exhibiting BDR in MMEF, whether alone or with BDR in FEV_1_. Our findings for those with BDR in FEV_1_ and MMEF confirm previous studies using similar BDR criteria for FEV_1_ [29, 30]. The similar baseline FEV_1_ and FVC seen in groups 1 and 2, with BDR in FEV_1_ only seen in group 1, highlights that the differences in improvements could indicate improvement in different regions of the lung. However, only a comprehensive study would confirm this.

In the current study, patients in group 1 were younger than groups 2 and 3, which may explain why positive BDR in FEV_1_ was not observed in groups 2 and 3. However, previous studies found no associations between age and BDR in FEV_1_ [6, 30] even after adjusting for baseline FEV_1_ [29]. The reason for this difference is unclear but could be due to the diagnostic criteria for COPD as previous studies used post-BD FEV_1_/FVC < 70% to define AO, while the current study used the LLN (i.e., z-score < -1.645). The smaller and focused sample size in the present study may reflect this difference.

Higher BMI was found in patients with BDR in FEV_1_ and MMEF than those without BDR in either measure, as in previous studies [8, 30]. Whether this reflects a weight-related effect on spirometric measures or a marker of deteriorating health and lung function is complex and requires prospective study of this interaction to explore putative reasons.

Smoking history, including smoking status, pack-years and years since quitting, was not different between those with and without positive BDR in FEV_1_. This likely indicates that smoking exposure per se does not affect the BDR of FEV_1_ in COPD nor the BDR of MMEF alone, highlighting that smoking exposure cannot explain the differences in physiological improvements. Our findings are thus supportive of previous studies that smoking exposure did not predict the BDR in FEV_1_ [29, 30].

Small airway has been shown to play an important role in the pathophysiology of COPD [15–18, 31]. Pathological studies have shown that small airway damage and impairments preceded the development of emphysema and airflow obstruction [15, 16, 31]. Further, longitudinal studies of the most commonly used physiological measures of SAD (MMEF) have demonstrated that small airway impairment appeared to precede emphysema and airflow obstruction [17], and was associated with a higher likelihood of COPD development [18]. Recently, a cross-sectional study has demonstrated that low MMEF (suggestive of SAD) was highly prevalent in COPD [32]. These studies suggest that small airways should be considered when managing COPD. Studies have shown measures of SA (i.e. MMEF and oscillometry techniques indices) can demonstrate a BDR in COPD [20]. However, there is no consensus about which test is most sensitive in assessing SA. Therefore, the MMEF was selected as a test of SA in this study because it is routinely collected and has previously been shown to be a useful marker of SAD [17, 18].

In routine lung function, the inclusion of MMEF has been challenging because of the wide variation found in a non-characterized general population [33]. However, recent studies have demonstrated its utility, particularly in the early detection of COPD [17, 18] and an essential predictor of COPD development [18]. These studies indicate that MMEF is of value in selected patient group and to answer specific questions reflecting present and future disease phenotype. Therefore, the current study is a proof-of-concept to assess whether BDR in MMEF detect patients showing physiological improvements in the SA following a bronchodilator. A cut-off of 30% to define a BDR in MMEF was pragmatically chosen, due to its previous use in other studies [27, 28].

In COPD, BDR in MMEF has only been assessed in two studies [21, 22]. In the first, 24 COPD patients showed notable improvement of MMEF (mean % change of 21.3 and 19.3) after 200ug and 400ug of salbutamol, respectively [21]. Park et al. in the second study showed less improvement in MMEF (mean % change of 8.25) after administering 200ug salbutamol [22]. In both studies, MMEF demonstrated changes after bronchodilator administration, as in the present study. However, our study (by pragmatic design/definition) demonstrated larger changes in MMEF that were accompanied by a BDR response of FEV_1_ in some patients and was able to identify others with BDR in MMEF alone.

It is important to emphasise that post-BD MMEF changes demonstrated a greater variance than post-BD FEV_1_ changes, which is consistent with the study by Borril and colleagues [19]. This raises a challenge in the interpretation of such changes in MMEF in the BDR assessment. The criteria we used to determine improvement in MMEF have been proposed by others [27, 28] and are certainly consistent with BDR of FEV_1_ in group 1 and would thus indicate a real change also in group 2 although why this diverges from the FEV_1_ response is currently unknown. However, as previously stated, this definition identifies a group of COPD patients who have important physiological improvements, likely in the SA and hence, may reflect a phenotype that benefits from more peripheral bronchodilator deposition using ultra-fine particles inhalers, although the clinical benefit will require further specific study.

The strength of our study is it is the first to provide data about BDR in the SA in identifying physiological COPD phenotypes. However, it is important to recognize weaknesses. First, due to the study's retrospective design, the data were limited to those routinely quantified. Therefore, more comprehensive studies that incorporate symptoms and other physiological parameters will be required to determine the clinical relevance of the characterization and its treatment and prognosis. Second, although it is still utilized in clinical practice, the current study used the LLN we chose a BDR in MMEF representing a BDR in the SA because of its availability in routine physiological assessment. However, it is recognized that MMEF is a highly variable spirometric measure, and it is also, in part, dependent on FVC. Therefore, adjusting MMEF for lung volumes is recommended, which is accomplished by altering the flow before and after bronchodilators to compare them at the same volume (called iso-volume MMEF) [34, 35]. The adjustment was recommended because MMEF failed to show a significant change after bronchodilator administration when FVC changed [34, 35]. In our study, the % change of MMEF in group 2 was greater than 30%, suggesting that this concern was not a factor in the current study. In group 3, the median % change in FEV_1_ and FVC were 3.6 and 2.3, respectively; thus, some of these patients may experience a significant change in the iso-volume MMEF. However, this is unlikely to affect our conclusions that BDR in MMEF could identify a response that was not captured by FEV_1_. Third, as there is no agreed threshold for a BDR in MMEF, we pragmatically used a cut-off of 30% based on previous studies [27, 28]. Hence, future comprehensive studies should evaluate whether the 30% cut-off represent improvement related to the small airways and is the best threshold for identification of a clinically important response. Finally, the present study assessed patients at two-time points on the same day and cannot determine whether patients with BDR in MMEF represent a distinct or more constant clinical phenotype. Hence, long-term studies are needed to confirm the reliability of BDR in MMEF alone as a clinical phenotype in COPD and hence its implications.

## Conclusion

This cross-sectional study identified that positive BDR of the SA (using MMEF) is common in COPD, and is present in all patients exhibiting BDR in FEV_1_, supporting the potential utility of MMEF in BDR assessment. BDR of the SA is also seen in some COPD patients even in the absence of BDR in FEV_1_, which may identify a group of patients that have a different pathophysiology, prognosis and potential treatment strategy. Therefore, assessing BDR using MMEF may play a role in the management of COPD.

## Supplementary Information

**Additional file 1. **Flowchart of the participants in study using the 0.70 criteria.

**Additional file 2: Supplementary Table 1.** Comparison of prevalence of BDR in MMEF in COPD patients using LLN criteria and 0.70

## Data Availability

The lung function data used and evaluated during the present study can be made available from the corresponding author, Prof. Elizabeth Sapey, upon reasonable request.

## Abbreviations

COPD: Chronic obstructive pulmonary disease
SAD: Small airways dysfunction
SA: Small airways
MMEF: Maximal mid-expiratory flow
FEV_1_: Forced expiratory volume in 1 s
FVC: Forced vital capacity
FEV_3_: Forced expiratory volume in 3 s
BDR: Bronchodilator response
BD: Bronchodilator
BMI: Body mass index
MMEF: Maximal mid-expiratory flow
SABA: Short-acting beta-2 agonist
SAMA: Short-acting muscarinic antagonist
ICS: Inhaled corticosteroid
LABA: Long-acting beta-2 agonist
LAMA: Long-acting muscarinic antagonist
GOLD: Global Initiative for Chronic Obstructive Lung Disease
