# Supplementary material for: The prevalence of bronchodilator responsiveness of the small airway (using mid-maximal expiratory flow) in COPD - a retrospective study
Source: BMC Pulm Med. 2022 Dec 30;22:493. doi: 10.1186/s12890-022-02235-0 (PMC9801537; doi:10.1186/s12890-022-02235-0)
Supplement: Supplementary file 1 — Additional file 1. Flowchart of the participants in study using the 0.70 criteria. [file 12890_2022_2235_MOESM1_ESM.docx]

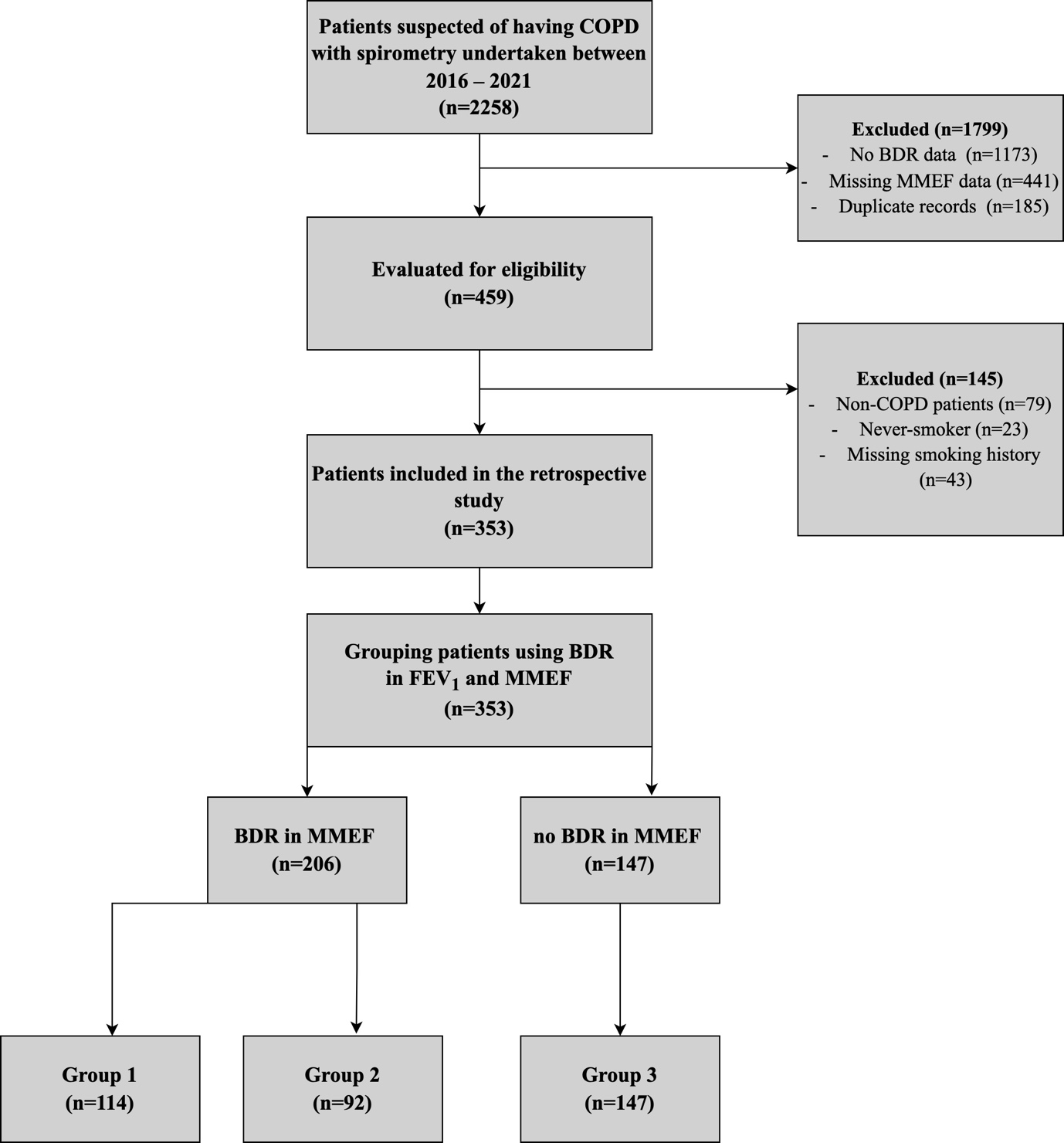


Flowchart of the participants in study using the 0.70 criteria.

**Legend:** This figure demonstrates the selection process for patients according to eligibility criteria. Group 1, those with BDR in FEV_1_ and MMEF; Group 2, those with BDR in MMEF alone; Group 3, those with no BDR in either FEV_1_ or MMEF.

**Abbreviations**: COPD, chronic obstructive pulmonary disease; MMEF, maximal mid-expiratory flow; FEV_1_, forced expiratory volume in 1 second; BDR, bronchodilator response.
