# Supplementary material for: The prevalence of bronchodilator responsiveness of the small airway (using mid-maximal expiratory flow) in COPD - a retrospective study
Source: BMC Pulm Med. 2022 Dec 30;22:493. doi: 10.1186/s12890-022-02235-0 (PMC9801537; doi:10.1186/s12890-022-02235-0)
Supplement: Supplementary file 2 — Additional file 2: Supplementary Table 1. Comparison of prevalence of BDR in MMEF in COPD patients using LLN criteria and 0.70 [file 12890_2022_2235_MOESM2_ESM.docx]

| **Supplementary Table 1. Comparison of prevalence of BDR in MMEF in COPD patients using LLN criteria and 0.70.** | | |
| --- | --- | --- |
| **Criteria for COPD diagnosis** | **FEV_1_/FVC** | |
|  | **LLN (n=314)** | **0.70 (n=353)** |
| **Group 1** | n=107 (34%%) | n=114 (32.3%) |
| **Group 2** | n= 79 (25%) | n= 92 (26.1%) |
| **Group 3** | n= 128 (41%) | n=147 (41.6%) |
| Group 1, BDR in FEV_1_ and MMEF; group 2, BDR in MMEF alone; group 3, no BDR in either measure. | | |
